# Seroconversion to *Lutzomyia intermedia* LinB-13 as a biomarker for developing cutaneous leishmaniasis

**DOI:** 10.1038/s41598-017-03345-0

**Published:** 2017-06-09

**Authors:** Augusto M. Carvalho, Kiyoshi F. Fukutani, Rohit Sharma, Rebecca P. Curvelo, José Carlos Miranda, Aldina Barral, Edgar M. Carvalho, Jesus G. Valenzuela, Fabiano Oliveira, Camila I. de Oliveira

**Affiliations:** 1 Instituto Gonçalo Moniz, FIOCRUZ, Salvador, Bahia 40296-710 Brazil; 2 Postgraduate Program in Health Sciences, Federal University of Bahia, School of Medicine, Salvador, Brazil; 3 Serviço de Imunologia do Hospital Universitário Professor Edgard Santos, Universidade Federal da Bahia, Salvador, Bahia Brazil; 4 Instituto de Investigação em Imunologia (iii), INCT, São Paulo, Brazil; 5 Vector Molecular Biology Section, Laboratory of Malaria and Vector Research, National Institute of Allergy and Infectious Diseases, National Institutes of Health, Rockville, Maryland USA

## Abstract

Sand flies inject saliva while feeding in the vertebrate host and anti-saliva antibodies can be used as biomarkers of exposure to *Leishmania* vectors. We expressed recombinant salivary proteins from *Lutzomyia intermedi*a, a vector of *Leishmania braziliensis*, and evaluated the seroreactivity in exposed individuals in search for exposure markers. We found a strong correlation among positive serology to recombinant proteins LinB-13, 26, 15, 21 and to salivary proteins: rLinB-13 was the top performing molecule; IgG4 was the most predominant antibody subclass and antibodies to rLinB-13 did not cross react with *Lu. longipalpis* salivary proteins. By evaluating a cohort of contacts of CL patients, we confirmed that rLinB-13, an antigen 5-related protein, is a marker of exposure to *Lu. intermedia* with high degree of accuracy. In a 5-year follow up, we determined that individuals who developed CL presented higher anti-rLinB13 IgG responses, before the appearance of clinical symptoms. They also presented a lower frequency of cellular responses to the parasite (DTH). Our results show that seroconversion to a salivary molecule, rLinB-13, is a marker of risk for CL development caused by *Leishmania braziliensis*. This highlight the possibility of developing tools based on vector molecules to manage the disease in endemic areas.

## Introduction

In the Americas, cutaneous leishmaniasis (CL) is caused mainly by *Leishmania braziliensis*, a parasitic protozoan transmitted by *Lutzomyia intermedia* sand flies. During *Leishmania sp*. transmission, the sand fly inoculates the vertebrate host with both *Leishmania* parasites and with salivary molecules. These molecules aid in blood feeding and some of these salivary proteins are immunogenic (rev. in ref. 1).

The humoral and cellular immune response to sand fly salivary proteins in animals including humans has been well documented (rev. in refs 2, 3) and the idea or concept of using sand fly salivary proteins as markers of vector exposure has been reported and used successfully for *Lutzomyia longipalpis* ^4–6^ and *Phlebotomus papatasi* ^7–10^. Therefore, the antibody response to salivary antigens can be used to probe for exposure to other sand flies and, hence, to assess the possibility of developing leishmaniasis associated to that sand fly species. However, the use of salivary gland sonicate (SGS) in large-scale epidemiologic assays is challenging due the difficulty inherent to sand fly capture or rearing, both necessary for salivary gland antigen preparation. Furthermore, SGS is a combination of various antigens, limiting therefore the purity or specificity of the antigen. To overcome this obstacle, transcriptomics and proteomics approaches have been effectively used in identifying and characterizing a number of sand fly salivary proteins (rev. in ref. 11). Sand fly salivary transcripomic approaches have been followed by the production of recombinant sand fly salivary proteins in heterologous systems and the use of these recombinant proteins for serologic testing. Sand fly recombinant salivary proteins LJM17 and LJM11 have been validated in the field as markers of exposure to *Lu. longipalpis* in Brazil^6, 12^ and the recombinant salivary protein PpSP32 has been successfully used as a marker for *Ph. papatasi* exposure in Tunisia and Saudi Arabia^13, 14^. These works underline the feasibility of using sand fly salivary recombinant proteins as epidemiological tools for vector exposure.

In addition to using anti-saliva antibodies to probe for sand fly exposure in animal reservoirs and humans, the anti-saliva immune response has also been associated with the risk of developing leishmaniasis upon infection with an infected sand fly^9, 15^. In Brazil, we observed that patients with CL caused by *Leishmania braziliensis* presented a significantly higher IgG response to *Lu. intermedia* SGS compared to individuals with subclinical *L. braziliensis* infection^8^ and natural exposure to *Lu. intermedia* bites indeed increases the risk of developing CL caused by *L. braziliensis* ^16^.

Based on the availability of the *Lu. intermedia* salivary gland transcriptome^17^, we expressed the most abundant salivary proteins from this sand fly species and searched for antigens that could act as exposure markers to *Lu. intermedia* in an area where CL is caused by *L. braziliensis*. Besides validating the top-performing antigen in a large cohort of household contacts, we also performed a 5-year follow up study of exposed individuals. We probed for serology to rLinB-13 and CL development and we show that individuals seropositive to a defined *Lu. intermedia* salivary recombinant protein are at increased risk of developing disease.

## Results

### Screening *Lu. intermedia* salivary proteins for markers of vector exposure

**Figure 1:**
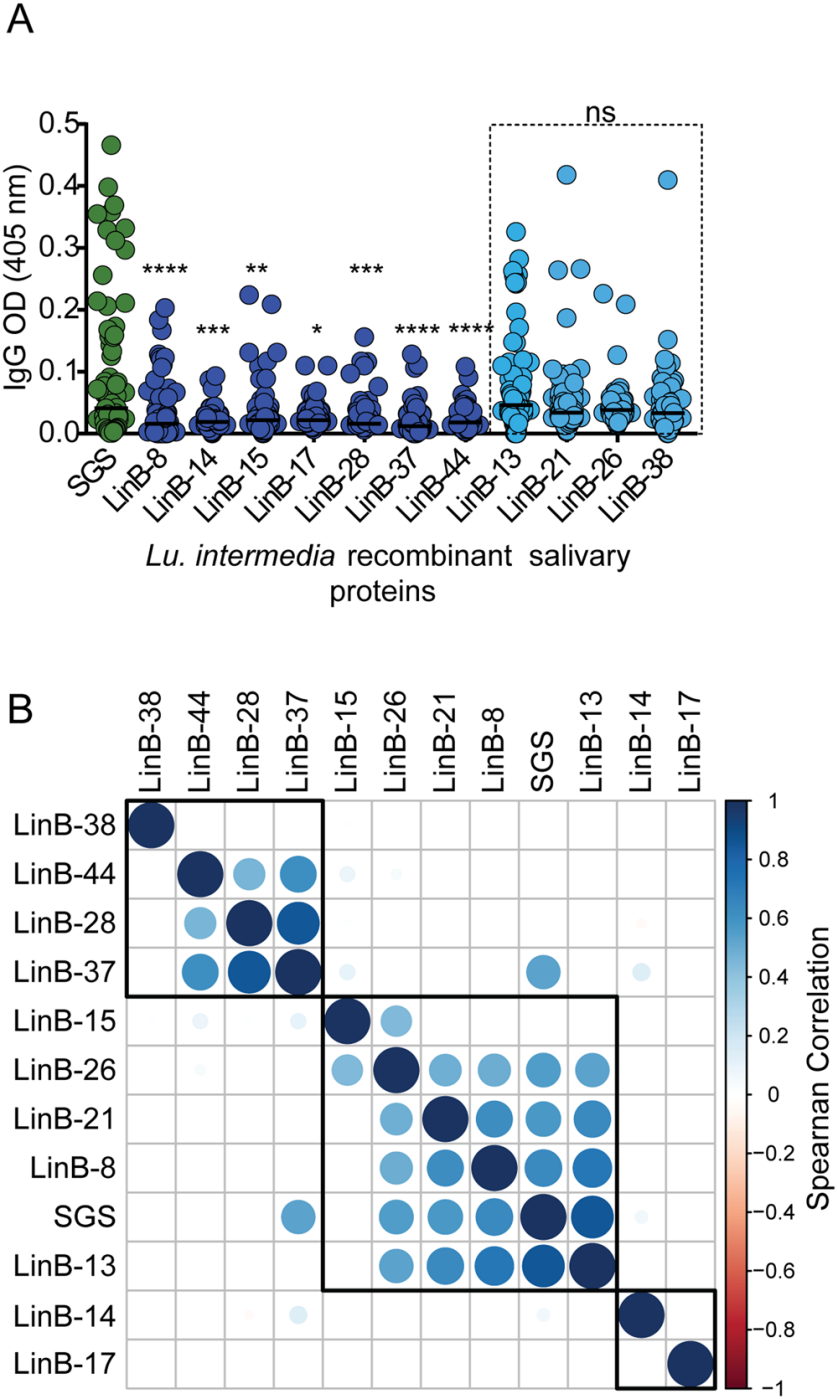
Screening *Lu. intermedia* salivary proteins for markers of vector exposure. (**A**) Total IgG response against *Lu. intermedia* SGS and recombinant salivary proteins was measured by ELISA in residents of a CL-endemic area (n = 89). Data are shown individually and black lines represent the median OD value for each antigen. All statistical analysis were performed in comparison to *Lu. intermedia* SGS. (**B**) Correlation matrix of IgG antibodies against *Lu. intermedia* SGS and recombinant salivary proteins in the same individuals represented in (**A**). Circles represent individual OD values, as shown in (**A**) Blue circles depict a positive Spearman correlation. Circle diameter and intensity of blue color depict higher Spearman r values. *P < 0.05; **P < 0.01; ***P < 0.001; ****P < 0.0001.

Eleven *Lu. intermedia* transcripts coding for secreted salivary proteins (LinB-8; LinB-13; LinB-14; LinB-15; LinB-17; LinB-21; LinB-26; LinB-28; LinB-37; LinB-38; LinB-44) were cloned and expressed as recombinant proteins in HEK cells (Table 1). *Lu. intermedia* recombinant proteins were tested with human sera (n = 89) from residents of a CL endemic area to determine IgG levels specific to these recombinant proteins (Fig. 1). We found a positive IgG response against all the recombinant proteins tested, however, only for LinB-13, LinB-21, LinB-26 and LinB-38 IgG levels were not statistically different to those detected against *Lu. intermedia* SGS (Fig. 1A). Using a correlation matrix to evaluate the response to *Lu. intermedia* SGS and the recombinant salivary proteins, we showed the presence of three major clusters: one cluster contained LinB-38, LinB-44, LinB-28 and LinB-37 and a second cluster contained LinB-14 and LinB-17, indicating that the IgG response to these proteins did not correlate to that observed for *Lu. intermedia* SGS (Fig. 1B). SGS was present in the third cluster together with LinB-8, LinB-13, LinB-15, LinB-21, LinB-26 salivary proteins confirming that the response to these salivary proteins is similar to that observed for *Lu. intermedia* SGS (Fig. 1A). Among these proteins, the response to rLinB-13 displayed the strongest correlation to SGS (*P* < 0.0001, r = 0.78) (Supplemental Table 1).

**Table 1:** Panel of *Lu. intermedia* recombinant salivary proteins.

Name	Accession Number	Weight (kDa)	Comment
LinB-8	KA660054	14.06	SP15 family member
LinB-13	KA660053	28.4	Antigen 5-related
LinB-14	KA660066	17.65	C-type lectin
LinB-15	KA660065	16.38	C-type lectin
LinB-17	KA660055	33.5	Similar to Factor Xa inhibitor
LinB-21	KA660057	44	Yellow related-protein
LinB-26	KA660060	22.9	30-kDa Phlebotomine
LinB-28	KA660061	13.8	SP15 family member
LinB-37	KA660070	15.41	ML domain salivary peptide
LinB-38	KA660071	12.34	10-kDa family member
LinB-44	KA660075	10.53	10-kDa family member

**Figure 2:**
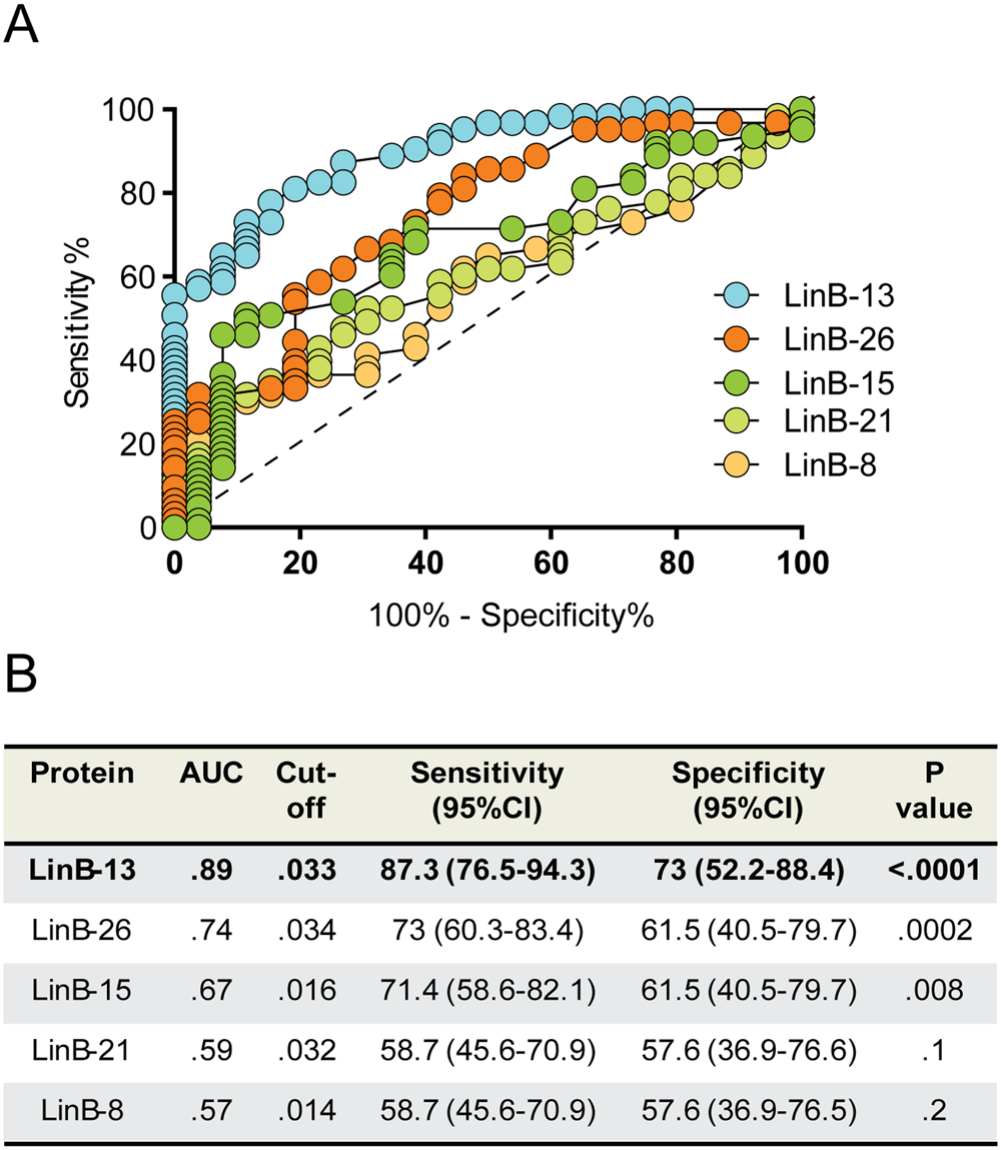
ROC antibody threshold level predicting ELISA positivity against *Lu. intermedia* SGS. (**A**) ROC curves were built using serology data from individuals seropositive (n = 63) or seronegative (n = 26) against *Lu. intermedia* salivary proteins. (**B**) Detailed information obtained from each ROC curve is shown: Area Under Curve (AUC), P values of the ROC curves, the cut-off values chosen, and sensitivity and specificity with the 95% confidence interval (CI).

We then evaluated whether these five recombinant proteins (LinB-8, LinB-21, LinB-15, LinB-26 and LinB-13) could predict seroconversion to *Lu. intermedia* SGS using a panel of seropositive (n = 63) and seronegative (n = 26) individuals. ROC analysis showed that the IgG response to LinB-13, LinB-26 and LinB-15 correctly discriminated individuals exposed to *Lu. intermedia* whereas LinB-21 and LinB-8 failed to do so (Fig. 2A,B). However, LinB-13 had a superior performance (AUC = 0.89, *P* < 0.0001) when compared to LinB-21 and LinB-8, displaying the highest sensitivity [87.3% (76.5–94.3)] and specificity [73% (52.2–88.4)], considering a cut off value of ﻿0.033 (Fig. 2B). These results show that, among the 11 recombinant proteins tested, LinB-13, was the top-performing molecule in predicting exposure to *Lu. intermedia* sand flies.

LinB-13 is a 28 kDa salivary protein, member of the family of antigen 5-related proteins, with ubiquitous distribution in sand flies [rev. in ref. 11]. Although its function is still unknown, it belongs to the cysteine rich secretory proteins, present in the saliva of most blood-sucking insects and in hookworms^18^. Multiple sequence alignment revealed that LinB-13 and homologues present in *Lu. longipalpis, Lu. ayacuchensis*, *Ph. duboscqi*, *Ph. papatasi*, *Ph. argentipes*, *Ph. perniciosus*, *Ph. orientalis, Ph. ariasi* and *Ph. sergenti* have a high degree of conservation at the amino acid level (Supplemental Fig. 1A). Phylogenetic analysis confirmed the clustering of LinB-13 and its homologues in *Lu. longipalpis* and *Lu. ayacuchensis* sand flies confirming the similarity between these molecules (Supplemental Fig.  1B).

### Validation of rLinB-13 as an exposure marker in a L. braziliensis transmission area

**Figure 3:**
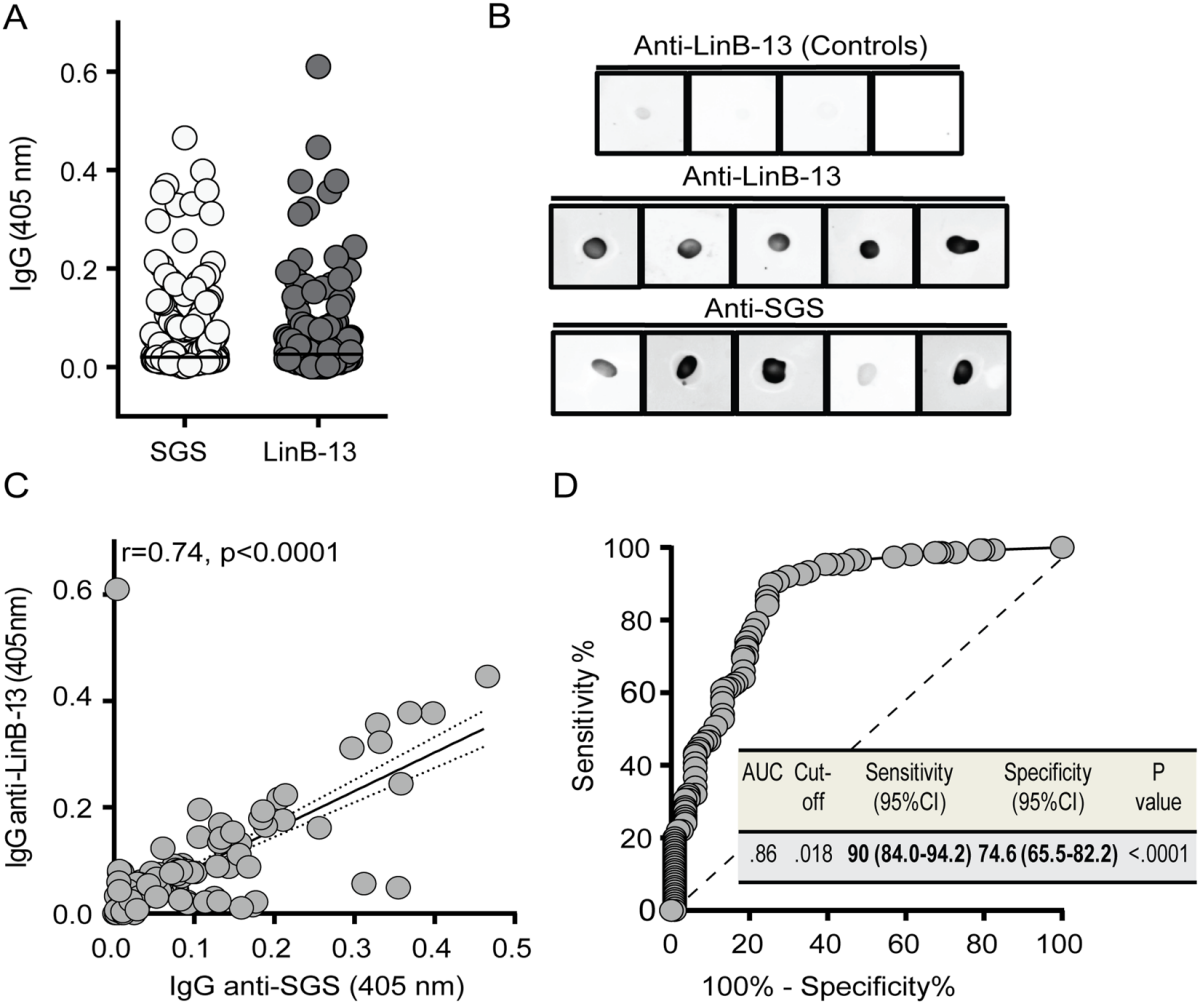
Validation of LinB-13 as a vector exposure maker in an area of *L. braziliensis* transmission. (**A**) Total IgG response against *Lu. intermedia* SGS and LinB-13 was measured by ELISA in household contacts (n = 264) of CL patients. Black lines represent the median OD value for each antigen. (**B**) Dot blot analysis against *Lu. intermedia* SGS and LinB-13 using serum samples from subjects with positive serology to SGS and to LinB-13 (n = 5) and from controls with negative serology (n = 4). (**C**) Spearman correlation between IgG to *Lu. intermedia* SGS and LinB-13 in the same individuals represented in (**A**). (**D**) ROC antibody threshold levels predicting positivity to LinB-13 and accompanying detailed information. Data are shown individually.

**Figure 4:**
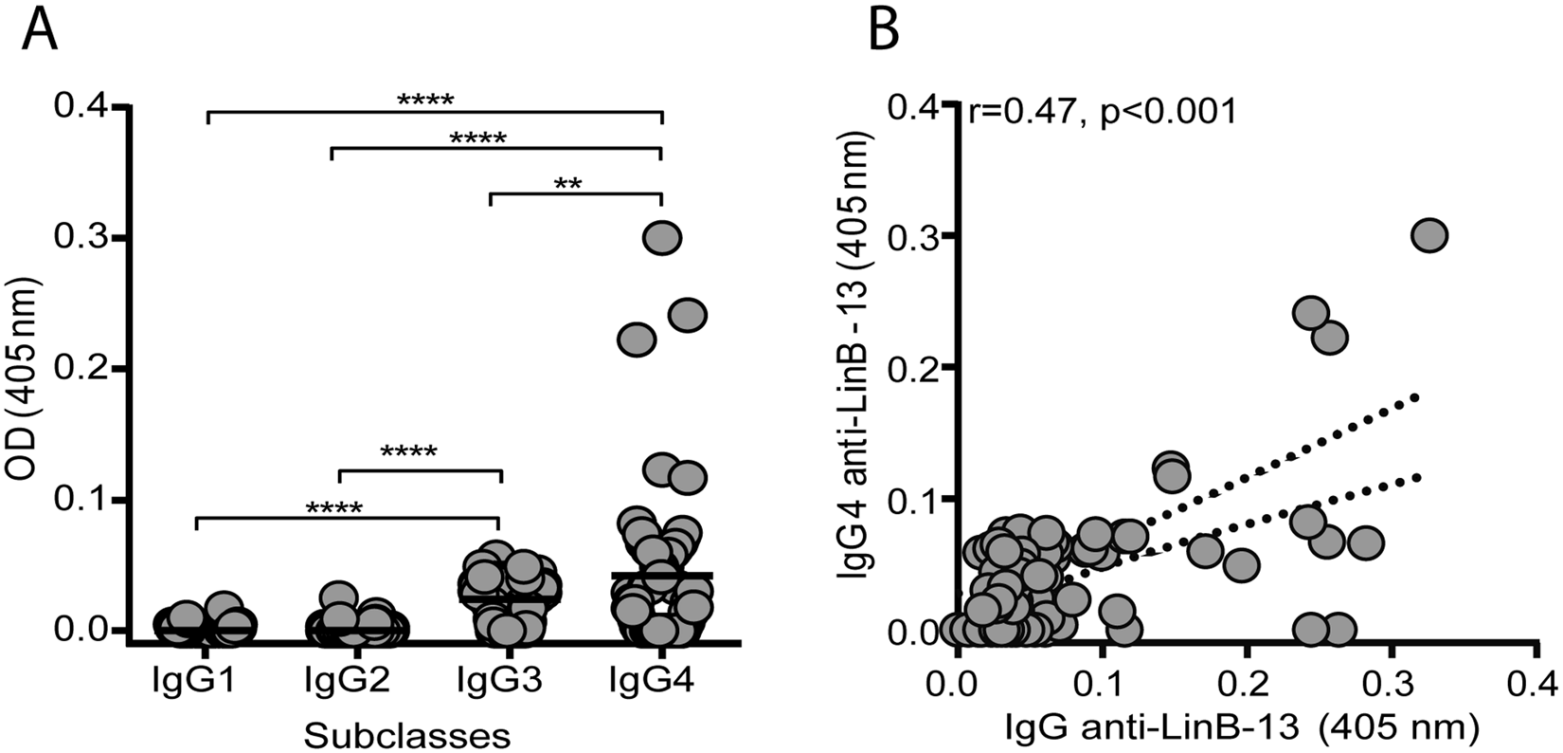
Anti-LinB-13 IgG subclasses detected in *Lu. intermedia*-exposed individuals. (**A**) IgG subclasses against LinB-13 were measured by ELISA using sera from residents of a CL-endemic area (n = 89). Black lines represent the median OD value for each antigen. (**B**) Spearman correlation between anti-LinB-13 IgG and IgG4 in the same individuals represented in (**A**). Data are shown individually **P < 0.01; ****P < 0.0001.

Next, we validated rLinB-13 as marker of exposure to *Lu. intermedia* by, evaluating the IgG response against this protein and against *Lu. intermedia* SGS. Among the household contacts (n = 264) of CL patients tested 136 (51.5%) had positive serology to rLinB-13 and 150 individuals (56.8%) had positive serology to SGS, indicating a similar degree of reactivity (Fig. 3A). Dot Blot analysis confirmed that rLinB-13 and *Lu. intermedia* SGS were equally recognized by individuals seropositive for both SGS and rLinB-13 but not by seronegative subjects (Fig. 3B). We also did not observe significant differences among seropositive or seronegative individuals to rLinB-13 regarding epidemiological characteristics such as age, gender, occupation, etc, (Supplemental Table 2). One exception was that subjects seropositive to rLinB-13 arrived more frequently at home after 4 pm. Furthermore, we found a strong correlation between IgG to rLinB-13 and to *Lu. intermedia* SGS (*P* < 0.0001, r = 0.74) (Fig. 3C) and ROC analysis confirmed, in this larger cohort, that antibodies against rLinB-13 discriminate a positive response to SGS (AUC = 0.86, *P* < 0.0001) (Fig. 3D). Therefore, rLinB-13 can predict exposure to *Lu. intermedia* with 90% sensitivity and 74.5% specificity (cut-off value of 0.018) (Fig. 3D). With regards to IgG subclasses, IgG4 was the most predominant isotype followed by IgG3 (Fig. 4A) and a significant correlation was found between anti-LinB-13 total IgG and anti-LinB-13 IgG4 (*P* < 0.001, r = 0.47) (Fig. 4B).

**Figure 5:**
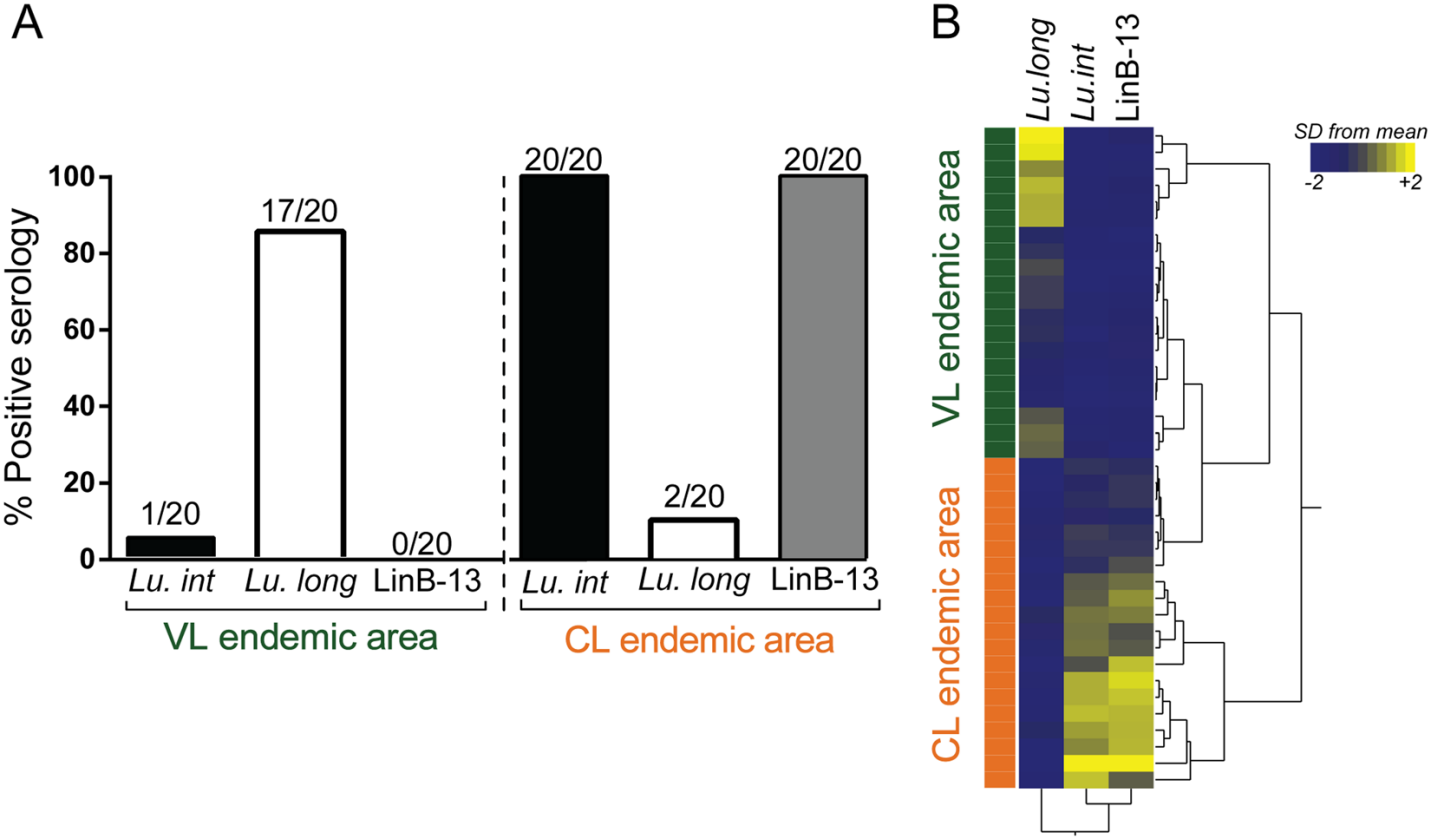
Cross-reactivity between serological responses to LinB-13 and to *Lu. longipalpis* saliva. Serum samples (n = 20) from residents of a CL endemic or of a VL endemic area were tested by ELISA against *Lu. intermedia* SGS, *Lu. longilpalpis* SGS and LinB-13. (**A**) Number of subjects with positive serology to each antigen, comparing the CL and the VL endemic areas. (**B**) Heat map depicting the pattern of *Lu. intermedia* SGS, *Lu. longilpalpis* SGS and LinB-13 recognition using Z-score normalized serology.

To evaluate cross-reactivity between anti-LinB-13 antibodies and IgG to salivary antigens from a related sand fly, we probed sera from individuals (n = 20) exposed to *Lu. longipalpis* against rLinB-13 and against *Lu. intermedia* SGS. Seventeen individuals (85%) presented positive serology to *Lu. longipalpis* SGS and one individual was seropositive to *Lu. intermedia* SGS but none of the sera reacted against rLinB-13 (Fig. 5A). On the contrary, 20 (100%) control sera (obtained from a CL endemic area) reacted against both *Lu. intermedia* SGS and rLinB-13 whereas two sera (10%) reacted against *Lu. longipalpis* SGS (Fig. 5A). Hierarchical clustering confirmed that the seroreactivity pattern of individuals from a CL endemic area is similar for both *Lu. intermedia* SGS and rLinB-13 whereas it is clearly distinct from that observed for individuals from a VL endemic area (Fig. 5B). These results highlight the potential of rLinB-13 to act as a marker of vector exposure in an area of *L. braziliensis* transmission.

### Antibodies to rLinB-13 as a risk marker of CL development

**Figure 6:**
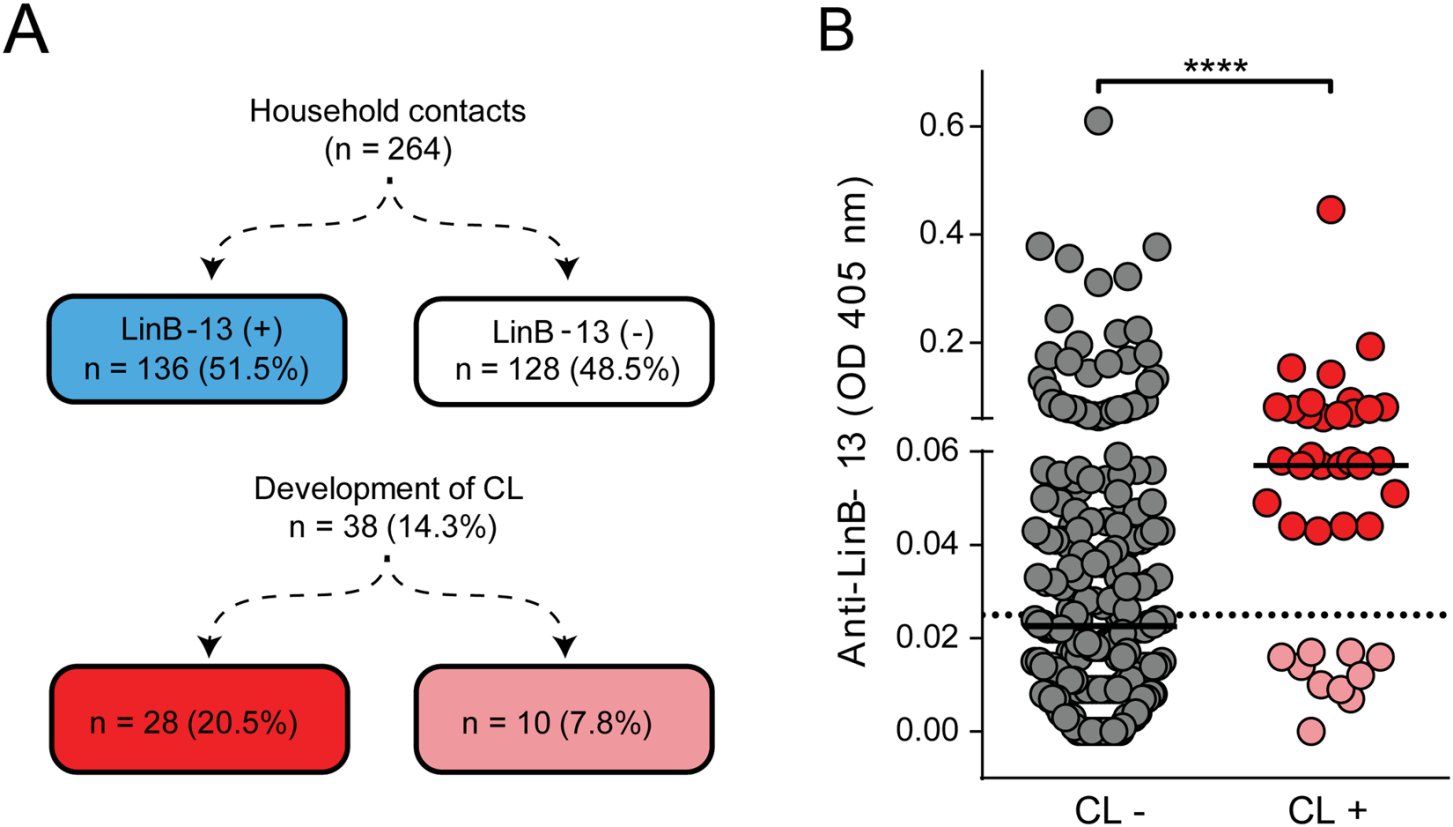
LinB-13 acts as risk factor for development of Cutaneous Leishmaniasis. (**A**) Total IgG response against LinB-13 was measured by ELISA in residents of a CL-endemic area who developed CL (n = 38) or who did not developed CL (n = 226). Data are shown individually. Black lines represent the median OD value for each antigen and dotted line represents the cut off value. (**B**) Flow chart showing the number of household contacts of CL patients who developed disease during the 5-year follow-up period, in both LinB-13 seropositive and seronegative individuals. ****P < 0.0001.

We previously showed that individuals with CL caused by *L. braziliensis* have a higher IgG response to *Lu. intermedia* SGS^8^ and this increases the risk of developing CL^16^. However, specific salivary molecule(s) within SGS that may play a role in this outcome remained unknown and we hypothesized that LinB-13 was a likely candidate based on its performance in predicting exposure to *Lu. intermedia*. We performed a 5-year follow up study in which 264 household contacts of CL patients were evaluated for presence of IgG to LinB-13. These evaluation were performed at the beginning of the study (Baseline) and at the 2^nd^ and 4^th^ years of follow-up. In parallel, individuals were evaluated yearly for the appearance of clinical signs of CL. At the time of enrollment in the study, we observed that 136/264 (51.5%) individuals developed anti-LinB13 antibodies whereas 128/264 (48.5%) did not (Fig. 6A). During the 5-year follow up, 38/264 (14.3%) subjects developed CL and, among these, 28/38 (73.7%) were seropositive to rLinB-13 whereas 10/38 (26.3%) were seronegative to rLinB-13 (Fig. 6A). In fact, the subjects who developed CL displayed a higher (p < 0.0001) anti-LinB-13 IgG response compared to those who remained free of disease. Also, the elevated anti-LinB-13 IgG response was detected before the development signs of the disease (Fig. 6B). Thus, individuals seropositive to LinB-13 are 2.6 times more likely to develop CL caused by *L. braziliensis* (RR = 2.6; 95% confidence interval = 1.3–5.2; *P* < 0.01) compared to seronegative subjects. Of note, seropositivity against LinB-13 did not change over time: individuals who presented IgG to LinB-13 at the time of enrollment in the study (Baseline) continued to display such response during the follow up period (Supplemental Fig. 2).

**Figure 7:**
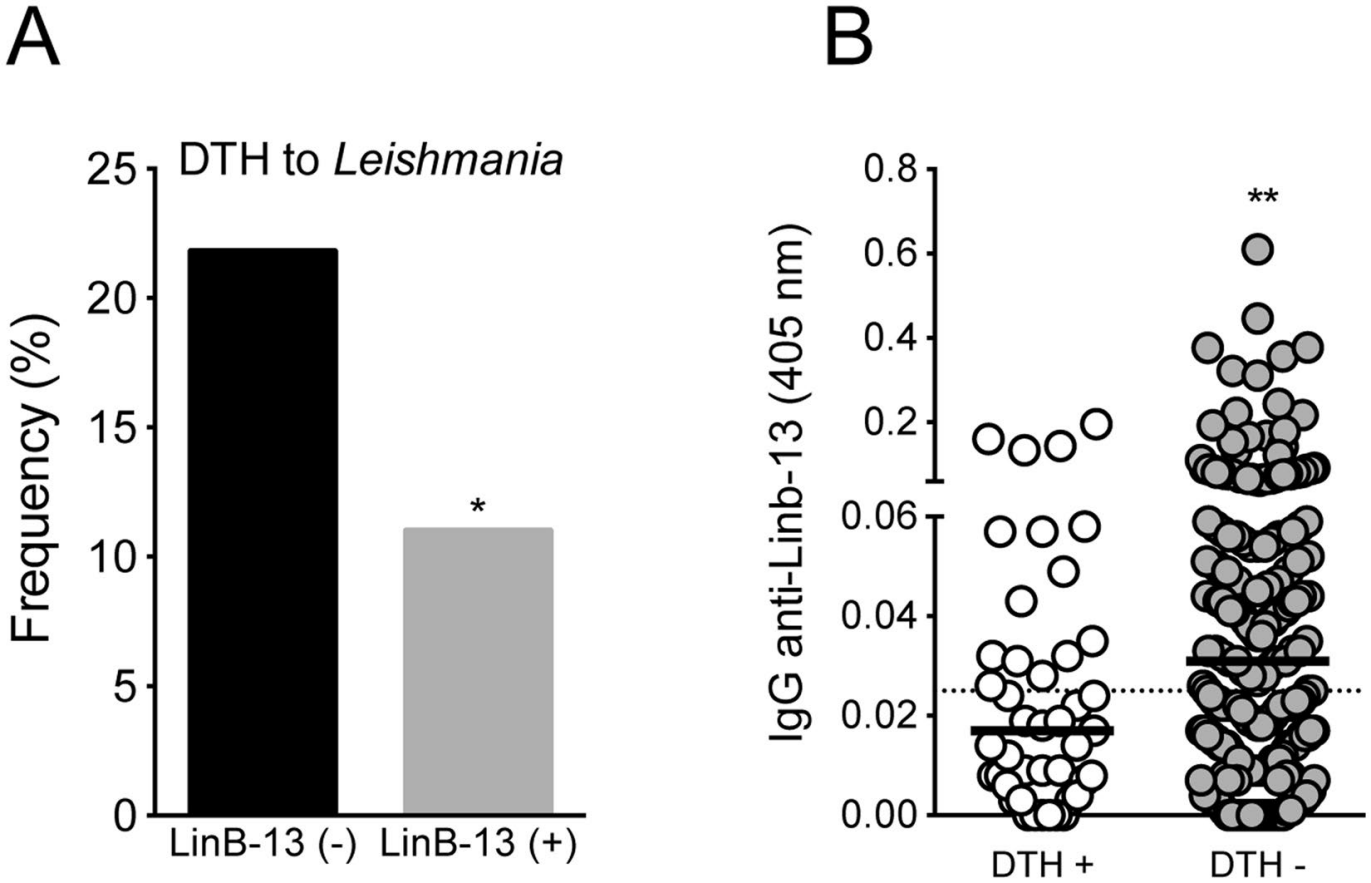
Lower response to *Leishmania* antigen in seropositive subjects to LinB-13. (**A**) Frequency of DTH reaction in subjects with positive or negative serology to LinB-13. (**B**) Total IgG response against LinB-13 was measured by ELISA in residents of a CL-endemic area who had positive (n = 43) or negative (n = 221) DTH to *Leishmania*. Data are shown individually. Black lines represent the median OD values for each group of individuals. *P < 0.05; **P < 0.01.

Presence of a delayed type hypersensitivity (DTH) reaction to *Leishmania* antigen is associated with protection against infection^19^. Among those individuals who seroconverted to rLinB-13, we detected a lower frequency (p < 0.05) of DTH + subjects (Fig. 7A). A positive DTH is a marker of subclinical infection in areas of *L. braziliensis* transmission^20^ but we observed that DTH + individuals displayed lower levels (p < 0.01) of anti-rLinB-13 IgG (Fig. 7B). Therefore, we show that development of antibodies to rLinB-13 is not concurrent with development of a cellular response to *Leishmania*.

## Discussion

Antibody responses to antigens present in arthropod saliva have been explored as vector exposure monitoring tool^21–23^, including vectors of leishmaniasis^4, 5, 7, 24^. This task has been enormously facilitated by the characterization and production of recombinant salivary proteins. In the present work we searched for immunodominant antigens in *Lu. intermedia* saliva using a panel of sand fly recombinant salivary proteins. We further validated the top-performing molecule in household contacts of CL patients and determined whether exposure to rLinB-13 could be used as a risk assessment tool.

Following the initial screening to identify the immunodominant molecules within *Lu. intermedia* salivary proteins, we found a strong correlation among anti-LinB-13 and anti-*Lu. intermedia* SGS antibodies, a result corroborated by ROC curve analysis. Similarly, antibody responses to LJM11 and LJM17^12^ also correlated with reactivity to *Lu. longipalpis* SGS, albeit with an inferior performance compared to rLinB-13. Besides LJM11 and LJM17 present in *Lu. longipalpis*, PpSP32 present in *Ph. papatasi* SP32^13–15^, SP03B present in *Ph. perniciosus* ^25, 26^ and rPorSP24 present in *Ph. orientalis* (rPorSP24)^27^ were identified as immunodominant salivary antigens and validated as markers of vector exposure in different endemic areas. In this work we identified rLinB-13 as a marker of *Lu. intermedia* exposure, a salivary antigen different in sequence and size to any other marker of exposure described so far. rLinB-13 was validated as a marker of sand fly exposure in a large cohort of individuals residing in a *L. braziliensis* transmission area.

As seen with *Lu. intermedia* SGS^16^ and in a study conducted with *Ph. papatasi*-exposed individuals in Egypt and Jordan, authors also found IgG4 to be the dominant subclass^28^. In the later, there was a positive correlation between concentrations of IgG4 and IgE, in agreement with reports showing concurrent IgE and IgG4 responses following exposure to *Aedes sp*.^29, 30^. Chronic exposure to *Aedes sp*. bites induces higher IgG4 antibodies against salivary proteins compared to other IgG subclasses^30^ and it has been suggested that IgG4 is a marker of intense exposure to arthropod vectors^31^. B-cell class switching to IgG4 and IgE is induced by IL-13^32^ but IL-10, in the presence of IL-4, can also drive IgG4 class switch in the absence of IgE^33^. Herein we have not addressed the presence of anti-LinB13 IgE but both IL-10 and IL-13 were detected upon stimulation of peripheral blood cells from *Lu. intermedia* exposed individuals with SGS^16^, suggesting a mechanistic explanation for the presence of IgG4 antibodies in *Lu. intermedia*-exposed individuals.

Besides the possibility of using sand fly salivary proteins to measure vector exposure, these molecules have also been employed to access the risk of disease development. In Tunisia, individuals who developed CL caused by *L. major* displayed significantly higher antibodies levels against *Ph. papatasi* saliva compared to subjects that remained disease-free^9^. In Turkey, patients with *L. tropica* lesions also showed higher levels of IgG against *Ph. sergenti* saliva compared to control individuals^7^. We corroborated these findings in Brazil: patients with CL caused by *L. braziliensis* presented a higher IgG response to *Lu. intermedia* SGS compared to individuals with subclinical *L. braziliensis* infection^8^, indicated by a positive DTH to *Leishmania*, in the absence of clinical signs. Indeed, higher serology to *Lu. intermedia* SGS increases the risk of developing CL^16^. These studies were performed using SGS and the specific salivary molecule or molecules associated with such outcome remained unknown. By following up individuals living in a CL endemic area and constantly exposed to *Lu. intermedia* we detected seroconversion to rLinB-13 before the appearance of clinical signs of CL. Importantly, positive serology to rLinb-13 was higher individuals who later developed CL, showing that seroconversion to rLinB-13 increases the risk of developing disease. This increased risk of disease was paralleled by a significantly lower frequency of a positive DTH response to *Leishmania* in seropositive subjects, suggesting that exposure to *Lu. intermedia* LinB-13 may negatively impact a cellular responses to the parasite. We previously showed that individuals exposed to *Lu. intermedia* present a higher frequency of CD4^+^CD25^+^IL-10-secreting cells and, *in vitro*, such response enabled parasite replication^16^. We speculate that LinB-13 may induce a similar response thereby promoting a parasite-friendly environment at the moment of transmission. However, we cannot rule out the hypothesis that a higher antibody response to LinB-13 reflects an increased exposure to *Lu. intermedia* and, in consequence, and increased probability of contact with infected sand flies and increased risk of developing CL. Since we do not know when infection occurred in subjects who presented CL, the latter is difficult to be addressed in human studies.

Experimental studies addressing how exposure to sand fly saliva could promote protection from leishmaniasis^34–37^ enabled the formulation of the following hypothesis: exposure to sand fly salivary proteins primes the development of CD4^+^IFN-γ-secreting T cells that, upon the bite of an infected sand fly, are rapidly mobilized to the inoculation site leading to macrophage activation and parasite killing (rev. in ref. 2). Our results, however, suggest that this response may not occur as such in areas of *L. braziliensis* transmission by *Lu. intermedia*, especially given our finding that a positive serology to LinB-13 is dissociated from DTH conversion to *Leishmania*. In Saudi Arabia^15^ and in Tunisia^38^, higher serology to salivary antigens has been reported in CL patients also suggesting a possible detrimental effect of exposure to sand fly saliva on human cutaneous leishmaniasis.

Sand fly salivary proteins are useful and practical tool to understand the dynamics of leishmaniasis transmission. Herein, we identified a single salivary molecule in *Lu. intermedia* saliva, LinB-13, that accurately predicts exposure to sand flies in a CL endemic area. Moreover, antibodies to rLinB-13 are a biomarker of risk of developing leishmaniasis caused by *L. braziliensis*. Host factors have been associated with susceptibility to CL^39–41^ and our study now identifies a sand fly salivary component that may also be decisive in the outcome of disease. We thus envisage a translational approach in which clusters of individuals with high titers of LinB-13 could be targeted for Insecticide Residual Spraying interventions, especially in areas where *L. braziliensis* transmission is peridomiciliar.

## Methods

### Study design and selection of individuals

This study was conducted in Corte de Pedra, municipality of Tancredo Neves, BA, Brazil, an area of *L. braziliensis* transmission by *Lu. intermedia* and it consisted of three phases (Supplemental Fig. 3). In Phase 1, we screened *Lu. intermedia* recombinant salivary proteins (n = 11) using sera (n = 89) from residents of the endemic area. In Phase 2, we validated the top-performing *Lu. intermedia* salivary molecule, as indicated by ROC analysis, in a larger cohort of household contacts of CL patients (n = 264). We also evaluated cross-reactivity of LinB-13 against sera from individuals residing in a VL endemic area (n = 20) (Vila Nova and Bom Viver), municipality of Raposa, São Luis, MA, Brazil^24^. In Phase 3, we evaluated whether exposure to LinB-13 is a risk marker for CL development. We tested sera from the cohort of household contacts of CL patients (n = 264). The cohort was established in 2010 and followed up to January 2015^42^. Inclusion criteria consisted of a negative history of any type of *Leishmania* infection, established after a medical interview and physical examination for signs consistent with active or previous CL or ML. At the moment of enrollment, blood was collected for serological assays against LinB-13. At the same time, the *Leishmania* Skin Test (DTH) was performed, as described below. The serological surveys were repeated at the 2^nd^ and 4^th^ year of follow up. Among the 264 household contacts, 237 were evaluated at the 2^nd^ year and 224 were evaluated at the 4^th^ year of follow up. The individuals not evaluated either developed CL or refused to participate. During follow-up, one visit was performed annually for active monitoring of CL development and subjects suspected of having CL were diagnosed by parasite isolation or by a positive PCR for *L. braziliensis*. This research was conducted with the approval of the Ethical Committee of the Hospital Prof. Edgard Santos (Salvador, Bahia, Brazil; 240/2009) and Comissão Nacional de Ética em Pesquisa (CEP, Brazilian National Ethics Committee, Brazil). Informed consent was obtained from each participant. All methods were performed in accordance with the guidelines and regulations determined by CEP.

### Leishmania Skin Test

The Leishmania Skin Test was performed at the moment of enrollment in the study and at the 2^nd^ and 4^th^ years of follow up. For skin test, 0,1 mL (15 μg/mL) of Soluble Leishmania Antigen, prepared as described elsewhere^43^, was injected intracutaneously on the volar surface of the forearm, and the greater diameter of induration was measured 48–72 h later. Induration of ≥ 5 mm was defined as a positive reaction.

### Sand flies and preparation of SGS

Adult *Lu. intermedia* or *Lu*. *longipalpis* sand flies were captured in Corte de Pedra and Cavunge, BA, Brazil, respectively. Sand fly identification and Salivary Gland Sonicate (SGS) preparation were performed as described elsewhere^44^.

### Cloning and expression of *Lu. intermedia* salivary transcripts

Eleven *Lu. intermedia* cDNA coding for secreted proteins (LinB-8, LinB-13, LinB-14, LinB-15, LinB-17, LinB-21, LinB-26, LinB-28, LinB-37, LinB-38, LinB-44) (Table 1)^17^ were amplified by PCR, cloned into VR2010-TOPO and sequenced as described^45^. Recombinant proteins were produced by transfection of 293-F cells (Invitrogen) with VR2010-TOPO plasmids coding for different *Lu. intermedia* salivary proteins by the Protein Expression Laboratory at the Frederick National Laboratory for Cancer Research (Frederick, MD). High-performance liquid chromatography purification of His-tagged *Lu. intermedia* salivary proteins was performed as described^6^ using an NGC Chromatography System (BioRad). Eluted proteins were detected at 280 nm and collected every minute on a 96-well microtiter plate using a BioFrac Fraction Collector (BioRad). Recombinant proteins were tested for endotoxin using a QCL-1000 LAL assay (Lonza). Endotoxin-free recombinant proteins were used in all assays. Aliquots of eluted proteins were checked by SDS-PAGE developed by silver staining. Edman sequencing of the N-terminus region of the purified recombinant proteins was performed at the Protein Chemistry Section, Research Technology Branch, NIAID, NIH (Rockville, MD). Proteins were stored at −70 °C until use.

### Analysis of anti–*Lu. intermedia* SGS antibodies and anti–*Lu. intermedia* salivary proteins antibodies

Humoral (IgG and IgG subclasses) response to *Lu. intermedia* SGS and to recombinant salivary proteins was determined as described^16, 46^. Recombinant proteins were used at 1 µg/ml. In all ELISA assays, the cut-off value was established employing sera from healthy volunteers (n = 30) from a non-endemic area and was determined as the mean optical density (OD) value plus 2.5 standard deviations.

### Dot Blot Analysis

Dot blot analysis was performed by spotting 1 µg of rLinB-13 or *Lu. intermedia* SGS onto nitrocellulose membrane (Invitrogen). Membranes were blocked with 5% BSA in PBS-T (0.1% Tween 20) for two hours. Serum samples (1/100) from individuals (n = 5) with positive serology for both LinB-13 and *Lu. intermedia* SGS or from non-exposed subjects (n = 4) were diluted in PBS-T with 0.1% BSA and incubated with spotted membranes for 45 minutes. Following another round of washing, anti-human HRP-conjugated IgG (Sigma) was added (1/20000) and membranes were incubated for 30 minutes. Signal detection was carried out using ECL system (Amersham) on an Image analyzer (ImageQuant Las 4000).

### Statistical analysis

Comparisons between 2 groups were performed by Mann–Whitney test and among 3 or more groups by Kruskal–Wallis test followed by Dunn multiple comparison tests. Categorical data were compared using the Fisher exact test. The matrix based on non-parametric Spearman correlation was performed using R version 3.2.1 (R Foundation for Statistical Computing, Vienna, Austria). ROC analysis was used to establish sensitivity and specificity of recombinant proteins in predicting anti- *Lu. intermedia* SGS positivity. Correlation between IgG to LinB-13 and *Lu. intermedia* SGS or between IgG and IgG4 to LinB-13 were checked using non-parametric Spearman test. Hierarchical cluster analysis of Z-score normalized serology response to *Lu. intermedia* SGS, *Lu. longipalpis* SGS and rLinB-13 using the Ward’s method with bootstrap (100x) was performed using JMP Statistical Discovery (V. 12). Statistical analyses were conducted using Prism (V. 5.0) (GraphPad Software) and differences were considered significant when *P* < 0.05. The relative risk (RR) was calculated using the following formula: RR = I_1_/I_0_, where I_1_ is the incidence of CL in exposed individuals (seropositive to LinB-13 salivary protein), and I_0_ the incidence of CL in non-exposed individuals (seronegative to LinB-13 salivary protein).

## Electronic supplementary material

Supplementary material
