# Supplementary material for: Seroconversion to Lutzomyia intermedia LinB-13 as a biomarker for developing cutaneous leishmaniasis
Source: Sci Rep. 2017 Jun 9;7:3149. doi: 10.1038/s41598-017-03345-0 (PMC5466628; doi:10.1038/s41598-017-03345-0)
Supplement: Supplementary file 1 — Supplementary material [file 41598_2017_3345_MOESM1_ESM.doc]

Supplementary Material

Seroconversion to *Lutzomyia intermedia* LinB-13 as a biomarker for developing cutaneous leishmaniasis

Augusto M. Carvalho^1,2,4^, Kiyoshi F. Fukutani^1,2^, Rohit Sharma^1^, Rebecca P. Curvelo^1,2^, José Carlos Miranda^1^, Aldina Barral^1,2^, Edgar M. Carvalho^1,2,4^, Jesus G. Valenzuela^3^, Fabiano Oliveira^3^ and Camila I. de Oliveira^1,2^

**Supplemental Table 1**. R values of spearman correlation among recombinant salivary proteins and *Lu. intermedia* SGS.

| **Name** | **R value** | **P value** | |
| --- | --- | --- | --- |
| LinB-8 | .37 | | <.0001 |
| LinB-13 | .78 | | <.0001 |
| LinB-14 | .09 | | .36 |
| LinB-15 | .15 | | .14 |
| LinB-17 | .10 | | .30 |
| LinB-21 | .24 | | .02 |
| LinB-26 | .40 | | <.0001 |
| LinB-28 | .13 | | .19 |
| LinB-37 | .14 | | .16 |
| LinB-38 | .38 | | <.0001 |
| LinB-44 | .10 | | .34 |

**Supplemental Figure 1**. **LinB-13 is an antigen 5-related related protein present in sand fly saliva**. (A) Sequence alignment of LinB-13 from *Lu. intermedia* and orthologs present in different sand flies species. Black and gray shading represent identical and similar amino acids, respectively. (B) Phylogenetic tree showing the similarity of antigen 5-related related proteins in different sand fly species. Bootstrap value, 1000.

**Supplemental Table 2**. Demographic and epidemiologic features of individuals from a CL-endemic area,

| **Characteristic** | **LinB-13 IgG+**  **(n = 136)** | **LinB-13 IgG-**  **(n = 128)** | **P value** |
| --- | --- | --- | --- |
| Median age, Y (range) | 14 (2-58) | 16 (2-63) | .15 |
| Male sex, No. (%) | 66 (48.5) | 56 (43.7) | .46 |
| Occupation, No. (%) |  |  | .90 |
| Agriculture | 24 (17.6) | 20 (15.6) |  |
| Domestic | 33 (24.2) | 32 (25) |  |
| Student/others | 79 (58) | 76 (59.3) |  |
| Time living in endemic area, median Y (range) | 14 (2-53) | 14.5 (2-63) | .16 |
| Home arrival after 4 PM, No. (%) | 40 (29.4) | 13 (10.1) | .0001 |

**Supplemental Figure 2. Kinetics of the humoral response to *Lutzomyia intermedia* LinB-13.** Total IgG response against LinB-13 was measured by ELISA in residents of a CL-endemic area at the time of enrollment (Baseline) (n=264) and at two subsequent time points in the follow up period (2^nd^ year, n= 237, and 4^th^ year, n=224). Data are shown individually and black lines represent the median OD value for LinB-13. NS- not significant.

**Supplemental Figure 3. Study flow chart.** Phase 1: screening of *Lu. intermedia* recombinant salivary proteins (n=11) using serum samples (n=89) from individuals residing in an area prevalent for *L. braziliensis* transmission by *Lu. intermedia*. Phase 2: validation of LinB-13 in sera (n=264) from household contacts of CL patients from the same area. Evaluation of cross-reactivity against *Lutzomyia longipalpis* SGS using sera from a VL endemic area (n=20) and sera from a CL endemic area (n=20). Phase 3: prospective analysis of LinB-13 as a for risk marker for CL development. Study design details are described in methods.
